# Unusual initial presentation of childhood acute lymphoblastic leukemia as massive ascites and pleural effusion in post-COVID-19 setting: a case report

**DOI:** 10.1097/MS9.0000000000000140

**Published:** 2023-03-24

**Authors:** Oadi N. Shrateh, Afnan W.M. Jobran, Haneen Owienah, Rabee Adwan, Yasmin Dwikat, Mohammad Najajreh

**Affiliations:** a Al-Quds University-School of Medicine, Abu-Dis, East Jerusalem; b Radiology Department; c Infectious Department; d Pediatric Department; e Pediatric Hematology and Oncology Department, Al-Istishari Arab Hospital, Ramallah, West Bank, Palestine

**Keywords:** acute lymphoblastic leukemia, ascites, case report, COVID-19 infection, pleural effusion

## Abstract

**Introduction:**

Acute lymphoblastic leukemia (ALL) in children typically presents with nonspecific manifestations such as fever, fatigue, lethargy, joint and bone pain, and bleeding diathesis. Ascites and pleural effusion as an initial presentation of ALL, although described, is exceedingly rare. However, this unusual initial presentation becomes much rarer in the post-coronavirus disease 2019 (COVID-19) setting. Herein, we aim to highlight such a rare initial presentation of childhood ALL that warrants clinical attention.

**Case Presentation:**

Two months following a COVID-19 infection, a 3-year-old male patient presented to the hospital with severe abdominal distention associated with occasional dyspnea. Physical assessment revealed a critically ill and pale patient with a distended abdomen and decreased air entry on the right side of the chest. Laboratory testing showed pancytopenia. Imaging studies confirmed the presence of massive ascites and pleural effusion. Bone marrow aspiration revealed CD10-positive pre-B-cell ALL. The patient was treated with chemotherapy and achieved complete remission.

**Conclusion:**

Rare manifestations of relatively common diseases create a barrier to prompt and effective detection and medical intervention. Although ascites and pleural effusion are rare conditions in ALL children patients, the occurrence of these pathologies in this particular patient, especially following COVID-19 infection, is an exceedingly rare event.

Highlights

- Pleural effusions are a common feature of nearly all hematological malignancies during the course of the disease, but they only sporadically manifest in this way.
- Severe acute respiratory syndrome coronavirus 2 (coronavirus disease 2019) infection has a detrimental effect on the prognosis of cancer patients.
- Rarity of initial presentation of childhood acute lymphoblastic leukemia as synchronous massive ascites and pleural effusion in post-coronavirus disease 2019 setting.

## Introduction

Acute lymphoblastic leukemia (ALL) is a malignant transformation and proliferation of lymphoid progenitor cells in the bone marrow, blood, and extramedullary sites. The vast majority of ALLs occur in children’s age group with a percentage of 80% of all childhood leukemias. ALL is the most frequent childhood malignancy, with an overall estimated incidence of 34 cases per million individuals in the United States^1^,^2^. Although ALL occurs primarily as a de novo neoplasm, Down syndrome, Fanconi anemia, Bloom syndrome, neurofibromatosis type I, and ataxia telangiectasia have all been identified as genetic syndromes that predispose to a minority of ALL cases in the pediatric population. Furthermore, ionizing radiation exposure, pesticides, and certain viruses, including Epstein–Barr virus and HIV, have also been linked to a higher risk of childhood leukemia^3^. Pleural involvement with leukemic cells is a frequent finding at postmortem autopsy but is rarely emergent throughout life^4^. However, ALL was reported to cause an isolated unilateral pleural effusion as an initial presentation^5^. Besides that, ALL can present initially with ascites, pleural effusion, and confusion in the adult age group^6^.

This report highlights the unusual initial presentation of childhood ALL in a 3-year-old patient presented with massive ascites concomitant with pleural effusion following coronavirus disease 2019 (COVID-19) infection.

## Case presentation

**Figure 1:**
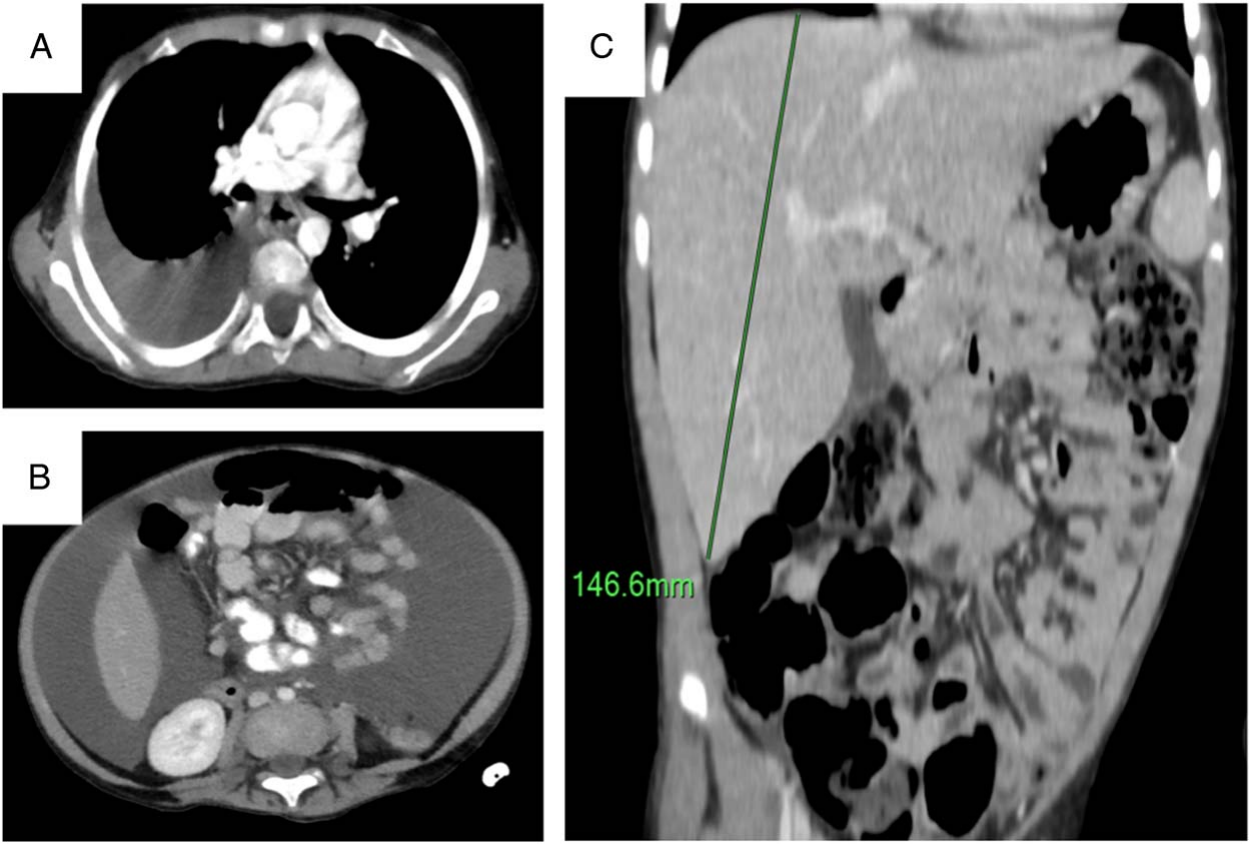
Computed tomography scan of (A) the chest showing right-sided pleural effusion, (B) the abdomen showing massive amounts of ascitic fluid, and (C) the abdomen showing hepatomegaly.

A.Z., a previously healthy 3-year-old male child was brought to the pediatric outpatient clinic with a 6-day history of nonproductive cough associated with fever and hypoactivity. One of his family members had a COVID-19 infection 1 week ago. The patient was tested for COVID-19 and was positive. The general medical condition of the child was significantly improved and the symptoms completely disappeared. Two months later, the patient came to the emergency department of the hospital with complaints of severe abdominal distention associated with occasional dyspnea. The patient had no fever, cough, abdominal pain, jaundice, or weight loss. He had no significant past medical, surgical, psychosocial, or drug history. No family history of genetic disorders. Upon admission, the physical assessment revealed a pale and critically ill child with multiple palpable submetacentric cervical lymph nodes. The heart sounds and jugular venous pressure were normal, but diminished breath sounds were noted at the level of the lung base on the right side. The abdomen was significantly distended with mild tenderness and a positive shifting dullness. The liver and spleen were not palpable. A purpuric rash was seen in the right lower quadrant region. Examination of lower limbs showed no pedal edema. Vital signs were normal except for a respiratory rate of 23 breaths per minute. No other remarkable findings were noted on examination. Laboratory evaluation was normal except for pancytopenia (Table 1). Imaging studies revealed the presence of massive ascites with mild liver enlargement and pleural effusion on the right side (Fig. 1a–c). No other prominent pathologic findings were seen. Analysis of pleural fluid demonstrated glucose of 90 mg/dl, protein of 3800 mg/dl, and lactate dehydrogenase of 235 IU/ml. In all, 1.5 l of the ascitic fluid were aspirated and subjected to biochemical and cytological assessment. This showed hemorrhagic content and prominent lymphocytic infiltrate with few atypical lymphocytes. Malignancy was suspected and bone marrow aspiration was performed. This, in turn, revealed more than 85% of bone marrow cells were lymphoblasts, and flow cytometry testing confirmed the diagnosis of CD10-positive pre-B-cell ALL. The patient was given intravenous fluid, fresh frozen plasma, platelets and started therapy according to the BFM-AIEOP (Berlin–Frankfurt–Munich – Associazione Italiana Ematologia Oncologia Pediatrica) 2009 protocol combined with methylprednisolone. The patient was also started on nystatin and trimethoprim–sulfamethoxazole. At the 3-month period of follow-up, lymphadenopathy was subsidized, and ascites and pleural effusion were significantly improved without recurrence. The patient achieved complete remission with less than 5% blasts in the bone marrow, a normal peripheral blood count, and no other symptoms of the disease. He also had no immunophenotypic evidence of minimal residual disease. According to the patient’s parents, their child showed good adherence and tolerability to the provided therapy without any noticeable adverse events.

**Table 1:** Laboratory results at the presentation

Laboratory parameter	The value	Reference range
Complete blood count		
Hb (g/dl)	7.1 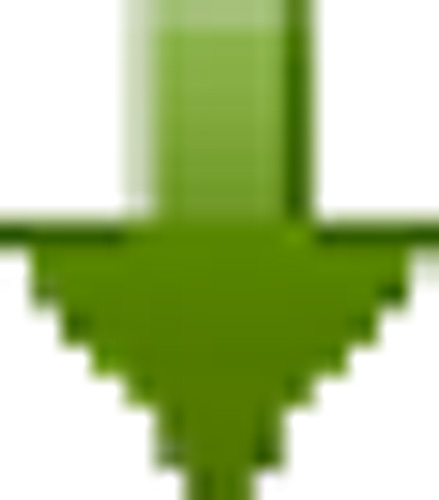	13.5–17.5
WBC (×10^3^)/µl	1.5 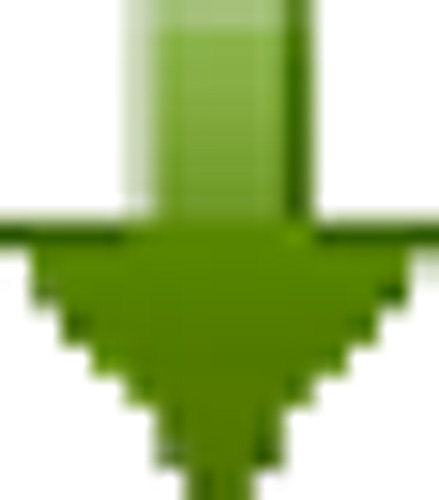	6–18
PLT (×10^3^)/µl	60 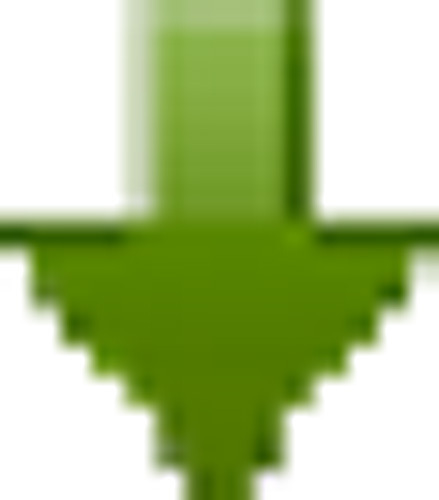	142–450
Inflammatory markers		
CRP (mg/dl)	0.7	0–6
ESR (mm/h)	4	0–15
LDH (IU/l)	157	135–225
Liver studies		
AST (U/l)	17	8–33
ALT (U/l)	21	7–55
GGT (U/l)	10	10–70
Total bilirubin (mg/dl)	1.2	1–1.2
Albumin (g/dl)	4.8	3.4–5.4
ALP (U/l)	162 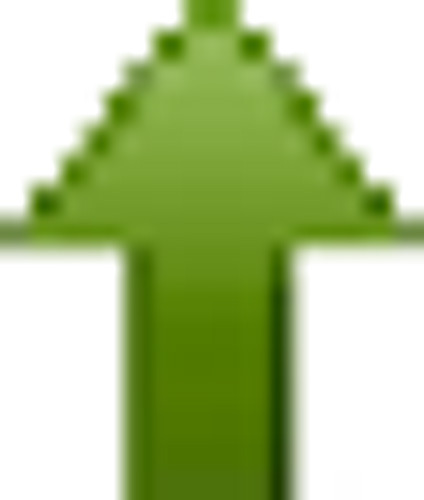	40–130
Kidney function		
CRE (mg/dl)	0.8	0.72–1.25
BUN (mg/dl)	14	5–18
UA (mg/dl)	3.8	3–7
Coagulation studies		
PT (s)	14.4	11–13.5
INR	1.1	1.1–1.2
Others		
Serum amylase	Normal	
Urinalysis	Normal	
Serum and urine electrolytes	Normal	
Blood cutlers	Negative	
Brucella serology	Negative	

ALP, alkaline phosphatase; ALT, alanine aminotransferase; AST, aspartate aminotransferase; BUN, blood urea nitrogen; CRE, creatinine; CRP, C-reactive protein; ESR, erythrocyte sedimentation rate; GGT, gamma-glutamyl transferase; Hb, hemoglobin; INR, international normalized ratio; LDH, lactate dehydrogenase; PLT, platelet; PT, prothrombin time; UA, uric acid; WBC, white blood cell.

## Discussion

All body organs and tissues are affected by leukemia, a disease that affects the entire body. Fever, pallor, and lethargic symptoms are frequent clinical manifestations. Rarely are acute leukemias with pleural infiltrations of malignant cells discovered during a person’s lifetime, but are a frequent discovery at autopsies^7^. Childhood acute leukemia, which accounts for one-third of all cases and has a fluctuating incidence rate of 10–45 cases per 106 children per year and a cumulative risk of 1 in 2000 up to the age of 15 years, is the most prevalent pediatric malignancy in developed cultures. ALL, the most prevalent form of pediatric leukemia, is an essentially fatal malignancy, as shown by a uniformly poor clinical prognosis prior to the development of effective therapy. To date, however, cure rates for ALL utilizing combination chemotherapy hover around 90%^8^, making this one of oncology’s true success stories.

There are numerous environmental factors that may be connected to ALL, but these links are frequently shaky, contradictory, or lack biological plausibility. A more conducive framework for studying this subject has been made available by large, multidisciplinary national research or multinational consortia^9^. At present, ionizing radiation is the only recognized causative factor for ALL, albeit in extreme situations^10^. By employing biological knowledge of cancer itself as the basis for developing, testing, and confirming hypotheses, it may be possible to understand the etiology of ALL most effectively.

Pleural effusions are a common feature of nearly all hematological malignancies during the course of the disease, but they only sporadically manifest in this way. This most frequently happens when the illness worsens, mostly in Hodgkin’s and non-Hodgkin’s lymphomas^11^. The rarity of childhood ALL manifesting as massive ascites and pleural effusion following COVID-19 infection in the literature^4^ is why this case deserves to be mentioned. When pleural fluid cytology is unable to identify the etiology of the effusion, closed biopsy or thoracoscopic biopsy is usually the next steps in the diagnosis of the fluid’s malignant origin. A total of 77% of malignant effusions with varied etiologies get positive results from these tests. Positive cytology is observed in 14–88% of individuals with lymphomatous effusions. However, the number of cancer cells in pleural specimens may be so low that even skilled cytologists cannot make a conclusive diagnosis^12^. As is highlighted in this research, a General Blood Picture in conjunction with a bone marrow biopsy can reliably confirm the diagnosis in all of these cases when a lymphoreticular condition is the underlying cause. Thus, a General Blood Picture must be performed on all individuals with effusions while the source of the condition is being investigated.

With mortality rates of over 20%, Severe acute respiratory syndrome coronavirus 2 (COVID-19) infection has a detrimental effect on the prognosis of cancer patients^13^. This is especially important in people with hematologic neoplasia and those undergoing allogeneic hematopoietic stem cell transplant, where the fatality rate exceeds 30%^14^. Four patients who are currently undergoing severe chemotherapy or immunotherapy as well as those with active illness are particularly at risk. This high mortality rate is a result of several variables, including advanced age, poor general health, and neutropenia, in addition to the disease itself. The mortality rates of the various hematologic neoplasia vary dramatically, with acute myeloblastic leukemia and lymphoproliferative disorders having the greatest mortality rates^15^. There is little available data on the prevalence and prognosis of COVID-19 infection in patients with ALL. Reports typically group patients with other hematologic malignancies due to the low prevalence of ALL in adulthood. One study on ALL found that patients with Philadelphia chromosome-positive (Ph+) ALL had a low incidence of COVID-19 infection. The study also suggested that tyrosine kinase inhibitors may be helpful in preventing patients from contracting the infection. This study was conducted in Italy during the first peak of the pandemic^16^.

## Conclusion

Ascites and pleural effusion can occur in approximately all hematological malignancies such as childhood ALL, during the disease process, but they only occur infrequently and rarely as the initial manifestations. This unusual and seldom presentation becomes much rarer during post-COVID-19 period.

## Ethical approval

Our institution has exempted this study from ethical review.

## Patient consent

Written informed consent was obtained from the patient for the publication of this case report and accompanying images. A copy of the written consent is available for review by the Editor-in-Chief of this journal on request.

## Sources of funding

The authors declare that writing and publishing this manuscript was not funded by any organization.

## Author contribution

O.N.S. and A.W.M.J.: writing the manuscript; H.O. and O.N.S.: imaging description; R.A., M.N., Y.D., O.N.S., and A.W.M.J.: reviewing and editing the manuscript.

## Conflicts of interest disclosure

The authors declare that there are no conflicts of interest regarding the publication of this article.

## Research registration unique identifying number (UIN)

1. Name of the registry: none.
2. Unique identifying number or registration ID: none.
3. Hyperlink to your specific registration (must be publicly accessible and will be checked): none.

## Guarantor

Oadi N. Shrateh.

## Provenance and peer review

Not commissioned, externally peer-reviewed.

## References

[1] Howlader N Noone AM Krapcho M . SEER Cancer Statistics Review (CSR) 1975–2014. National Cancer Institute; 2021.

[2] Siegel DA Henley SJ Li J . Rates and trends of pediatric acute lymphoblastic leukemia – United States, 2001–2014. MMWR Morb Mortal Wkly Rep 2017;66:950–954. 28910269 10.15585/mmwr.mm6636a3 PMC5657918

[3] Onciu M . Acute lymphoblastic leukemia. Hematol Oncol Clin North Am 2009;23:655–674. 19577163 10.1016/j.hoc.2009.04.009

[4] Dix DB Anderson RA McFadden DE . Pleural relapse during hematopoietic remission in childhood acute lymphoblastic leukemia. J Pediatr Hematol Oncol 1997;19:470–472. 9329473 10.1097/00043426-199709000-00013

[5] Mishra AK Kumar S Babu S . Rare initial presentation of ALL as pleural effusion. Egypt J Chest Dis Tuberc 2015;64:615–616.

[6] Roushan N Shahi F Mirzazadeh A . Acute leukemia presenting with ascites and confusion. Leuk Lymphoma 2007;48:1234–1236. 17577793 10.1080/10428190701258362

[7] Parkin DM Stiller CA Draper GJ . The international incidence of childhood cancer. Int J Cancer 1988;42:511–520. 3170025 10.1002/ijc.2910420408

[8] Inaba H Greaves M Mullighan CG . Acute lymphoblastic leukaemia. Lancet 2013;381:1943–1955. 23523389 10.1016/S0140-6736(12)62187-4 PMC3816716

[9] UK Childhood Cancer Study Investigators. The United Kingdom Childhood Cancer Study of exposure to domestic sources of ionising radiation: 1: radon gas. Br J Cancer 2002;86:1721–1726. 12087456 10.1038/sj.bjc.6600276 PMC2375400

[10] Preston DL Kusumi S Tomonaga M . Cancer incidence in atomic bomb survivors. Part III: Leukemia, lymphoma and multiple myeloma, 1950–1987. Radiat Res 1994;137(2 suppl):S68–S97. 8127953

[11] Berkman N Breuer R Kramer MR . Pulmonary involvement in lymphoma. Leuk Lymphoma 1996;20:229–237. 8624461 10.3109/10428199609051612

[12] Lossos IS Intrator O Berkman N . Lactate dehydrogenase isoenzyme analysis for the diagnosis of pleural effusion in haemato-oncological patients. Respir Med 1999;93:338–341. 10464900 10.1016/s0954-6111(99)90315-3

[13] Lee LYW Cazier J-B Starkey T . COVID-19 prevalence and mortality in patients with cancer and the effect of primary tumour subtype and patient demographics: a prospective cohort study. Lancet Oncol 2020;21:1309–1316. 32853557 10.1016/S1470-2045(20)30442-3 PMC7444972

[14] Vijenthira A Gong IY Fox TA . Outcomes of patients with hematologic malignancies and COVID-19: a systematic review and meta-analysis of 3377 patients. Blood 2020;136:2881–2892. 33113551 10.1182/blood.2020008824 PMC7746126

[15] Piñana JL Martino R García-García I . Risk factors and outcome of COVID-19 in patients with hematological malignancies. Exp Hematol Oncol 2020;9:21. 32864192 10.1186/s40164-020-00177-z PMC7445734

[16] Foà R Bonifacio M Chiaretti S . Philadelphia‐positive acute lymphoblastic leukaemia (ALL) in Italy during the COVID‐19 pandemic: a Campus ALL study. Br J Haematol 2020;190:e3–e5. 32368790 10.1111/bjh.16758 PMC7267647

